# SHI7 Is a Self-Learning Pipeline for Multipurpose Short-Read DNA Quality Control

**DOI:** 10.1128/mSystems.00202-17

**Published:** 2018-04-24

**Authors:** Gabriel A. Al-Ghalith, Benjamin Hillmann, Kaiwei Ang, Robin Shields-Cutler, Dan Knights

**Affiliations:** a Bioinformatics and Computational Biology, University of Minnesota—Twin Cities, Minneapolis, Minnesota, USA; b Computer Science, University of Minnesota—Twin Cities, Minneapolis, Minnesota, USA; c Biotechnology Institute, University of Minnesota—Twin Cities, Minneapolis, Minnesota, USA; University College Cork

**Keywords:** algorithm, bioinformatics, metagenomics, microbiome, pipeline, QC, quality control, sequencing, short read

## Abstract

Next-generation sequencing technology is of great importance for many biological disciplines; however, due to technical and biological limitations, the short DNA sequences produced by modern sequencers require numerous quality control (QC) measures to reduce errors, remove technical contaminants, or merge paired-end reads together into longer or higher-quality contigs. Many tools for each step exist, but choosing the appropriate methods and usage parameters can be challenging because the parameterization of each step depends on the particularities of the sequencing technology used, the type of samples being analyzed, and the stochasticity of the instrumentation and sample preparation. Furthermore, end users may not know all of the relevant information about how their data were generated, such as the expected overlap for paired-end sequences or type of adaptors used to make informed choices. This increasing complexity and nuance demand a pipeline that combines existing steps together in a user-friendly way and, when possible, learns reasonable quality parameters from the data automatically. We propose a user-friendly quality control pipeline called SHI7 (canonically pronounced “shizen”), which aims to simplify quality control of short-read data for the end user by predicting presence and/or type of common sequencing adaptors, what quality scores to trim, whether the data set is shotgun or amplicon sequencing, whether reads are paired end or single end, and whether pairs are stitchable, including the expected amount of pair overlap. We hope that SHI7 will make it easier for all researchers, expert and novice alike, to follow reasonable practices for short-read data quality control.

**IMPORTANCE** Quality control of high-throughput DNA sequencing data is an important but sometimes laborious task requiring background knowledge of the sequencing protocol used (such as adaptor type, sequencing technology, insert size/stitchability, paired-endedness, etc.). Quality control protocols typically require applying this background knowledge to selecting and executing numerous quality control steps with the appropriate parameters, which is especially difficult when working with public data or data from collaborators who use different protocols. We have created a streamlined quality control pipeline intended to substantially simplify the process of DNA quality control from raw machine output files to actionable sequence data. In contrast to other methods, our proposed pipeline is easy to install and use and attempts to learn the necessary parameters from the data automatically with a single command.

## Abstract (precis)

Quality control of high-throughput DNA sequencing data is an important but sometimes laborious task requiring background knowledge of the sequencing protocol used (such as adaptor type, sequencing technology, insert size/stitchability, paired-endedness, etc.). Quality control protocols typically require applying this background knowledge to selecting and executing numerous quality control steps with the appropriate parameters, which is especially difficult when working with public data or data from collaborators who use different protocols. We have created a streamlined quality control pipeline intended to substantially simplify the process of DNA quality control from raw machine output files to actionable sequence data. In contrast to other methods, our proposed pipeline is easy to install and use and attempts to learn the necessary parameters from the data automatically with a single command.

## INTRODUCTION

Next-generation sequencing (NGS) technology has become increasingly common across the biological sciences (1). The emergence of quality control (QC) software in tandem with the influx of NGS data highlights a need for measures to reduce noise, improve base call quality, increase read length, filter out spurious sequences, split sequencing lanes by barcode for pooled sequencing runs, and otherwise improve the signal-to-noise ratio present within the large volume of data used to drive downstream analyses and make important decisions.

However, the increasing number of sequencing protocols available can make it difficult for a nontechnical user to understand how to tune subtly different QC parameters when processing raw data. Different sequencing facilities use different techniques to shear longer DNA molecules into sufficiently short fragments for the sequencing instrument to process. Furthermore, different DNA preparation kits may be used and with different sequencing platform adaptors. There may be further points of difference as well, depending on the type of study performed; for instance, whereas shotgun sequencing methods attempt uniform coverage over all input DNA molecules, amplicon sequencing methods seek to minimize sequencing cost by targeted amplification of specific (marker) genes (2).

**FIG 1:**
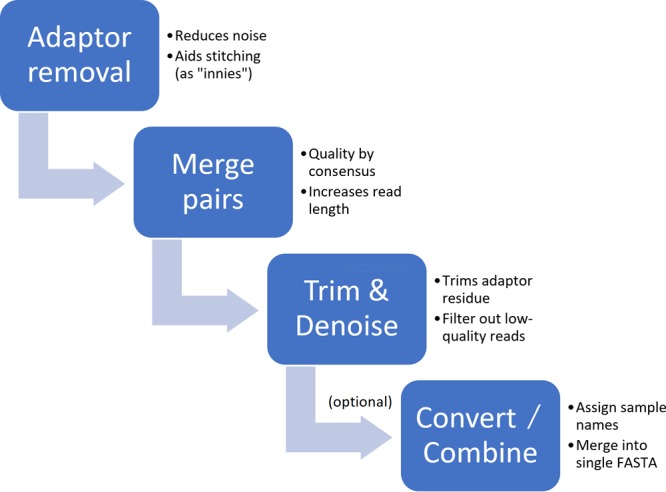
Linear schematic of the basic quality control procedure for marker gene (microbiome) data. The process flows from removing known technical artifacts, to assembling short contiguous regions, to trimming remaining contamination poststitching and creating a final set (or optionally, single pooled file) of sequences in the desired format (FASTA or FASTQ). Notable exceptions to this procedure exist: for instance, pairs may not be stitchable depending on the insert size for shotgun sequencing.

**Common workflows.** Despite numerous differences, the basic QC workflow for short-read sequencing has some common ground following sample preparation. (Fig. 1 shows a simplified schematic in context of microbiome sequencing.) Essentially, the result of a typical paired-end sequencing run results in one or more pairs of FASTQ files containing raw sequence information for 100- to 300-bp sequences along with quality scores representing the sequencing instrument’s measure of confidence in the accuracy of each base call. This is useful because a rudimentary quality control procedure may read these scores and determine, through a set of logical parameters, how much of each short read to retain. These scores may also be used directly by downstream applications to weight the influence of particular bases in alignments, such as in recent versions of the popular bowtie2 aligner (3). In some cases, there are multiple samples contained within a single sequencing lane, each with its unique sample barcode, as in multiplex marker gene sequencing (4), which subsequently must be demultiplexed in order for downstream analyses to differentiate among the various samples.

At an early stage in the QC process, it is essential to remove sequencing instrument adaptors introduced by the sequencing platform chemistry. Depending on the protocol used, some data may be more heavily contaminated with adaptors than others (5). The presence of adaptors can influence downstream analyses, including other QC steps, particularly if downstream global alignment (e.g., clustering) or end-to-end alignment (e.g., most short-read mapping) will be performed. Because these adaptor nucleotides will not be present in reference sequences or databases, their presence in the reads will decrease alignment scores. In some cases, paired-end sequencing protocols allow for the paired ends to overlap one another, allowing the two reads in a pair to be merged or “stitched” together to form a single, often longer or higher-quality contig. Throughout the region where both reads in a pair overlap, consensus quality determination is possible in cases of disagreement between pairs by retaining the higher quality of any two discordant bases. If the region of overlap is shorter than each individual read, this stitching also allows for the assembly of the two reads into a single longer contig. Merging of pairs hence both improves quality in the region of overlap and extends the read, both of which improve the accuracy of downstream analysis (6). After stitching, any remaining poor-quality regions, often located near the ends of the reads where the average base quality is lowest in general (6), can be trimmed until an acceptable quality is achieved throughout the read. Finally, these quality-controlled reads may be converted into the simpler FASTA format devoid of quality information, and in some domains, such as microbiome analysis, samples may be pooled into a single file with sequence headers indicating which biological sample a read came from, using standards-compliant formatting (7).

Each of these typical steps in QC has received extensive study, and there exist a variety of tools for performing these steps. Under the reasonable baseline assumption that any such tool has a profile of strengths and weaknesses, it is not our goal here to perform extensive meta-analyses thereof but instead to provide a user-friendly pipeline integrating a small number of well-known tools under a highly simplified interface. The primary contribution of our QC pipeline, SHI7 (pronounced “shizen”), is its ease of use. Specifically, SHI7 is trivial to install (either systematically with Conda, or as a portable standalone package with all dependencies included), easy to run from the command line, and features a learning module that makes data-driven predictions for various QC parameters and presents these predictions for the user to run directly or tweak as desired. Importantly, although we do not posit that SHI7 will outperform any dedicated tool(s) or pipeline(s) on any well-defined short-read sequencing workflow, it is expected to perform reasonably well with little user intervention or expert knowledge across various workflows and data sources, especially when knowledge of sequencing and DNA preparation methodology is scarce or unreliable.

## RESULTS

**FIG 2:**
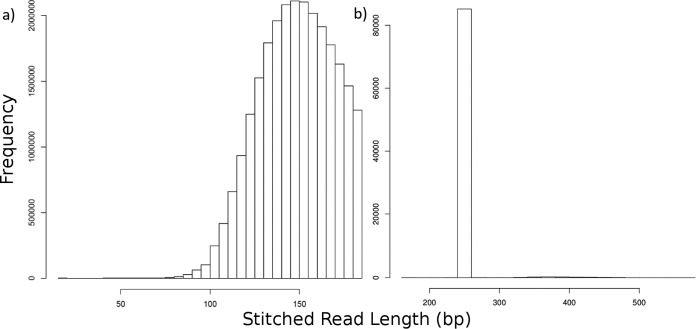
Histogram of stitched read lengths in a single Human Microbiome Project (HMP) metagenomic sample (a) and 16S V4 Primate Microbiome Project (PMP) sample (b). (a) A shotgun metagenomic sample produces stitched contigs spanning a range of lengths. The truncation after read lengths of 185 bp is due to enforcing a minimum overlap length of 15 base pairs, which in a data set consisting of 100-bp reads is the maximum allowable length (100 + 100 − 15). Because the mean of this distribution is 148.6 and its standard deviation is 20.62, the coefficient of variation (CV) is 0.139, above the 0.1 threshold under which the data would be considered amplicon-like by default; the data are hence considered shotgun reads by SHI7. (b) A 16S amplicon sample produces a distinct histogram marked by high representation of certain contig lengths corresponding to target gene size, in this case 252 and 253 base pairs, and a much lower CV (mean = 254.4, SD = 15.7; CV = 0.062). Most residual longer reads match PhiX174, an Illumina control contaminant, and are later removed by SHI7 in “learning mode” by filtering out sequences within a mean read length ± SD/2 in amplicon samples.

SHI7 was evaluated on publicly available sequence data from various sources, including the Human Microbiome Project (HMP) (8). A random deep shotgun sequencing sample was selected, for simplicity, from the associated HMP data in the Sequence Read Archive for analysis: SRS014271 (tongue). Without considering specifics of the sequencing platform, chemistry, adaptors, read lengths, library size, or paired-end status, the contents of the sequence archive were extracted into a new folder, and the file called “singletons” was removed. SHI7 determined that there was some “TruSeqv2” adaptor contamination, which it removed. It determined these were not amplicon reads, but stitchable shotgun reads (just over 60% of the reads in this sample could be stitched together, which it performed). The distribution of stitched lengths resembled a normal distribution centered around 150 base pairs (bp), as the command line debug output shows (Fig. 2a). The final trimming removed fewer than 0.1 bases, on average, from either end of the stitched reads, and the average base quality throughout was 36.3 (very high), with an average read length of just under 150 bp. Processing time was around 18 min for the 24-GB pair of FASTQ files on 16 cores of a Xeon E7-4850 server over gigabit network SATA storage.

**FIG 3:**
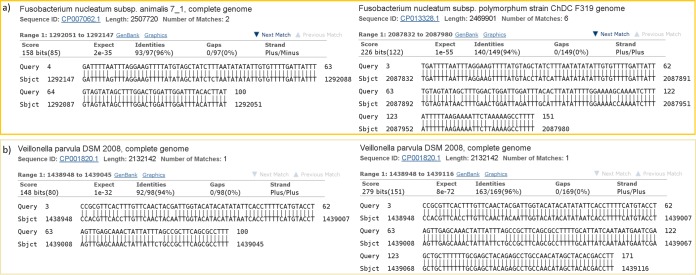
Comparison of illustrative BLAST alignments before and after SHI7 quality control on the same reads of an HMP shotgun sample. Panel a (top) shows the SHI7 QC read (right) achieving a different best-scoring alignment than the non-QC read (left) despite the former’s slightly lower identity (SHI7 alignment, 94% and E value of 1e−55; non-QC, 96% and E value of 2e−35). The same reference as in the non-QC alignment also appears for the SHI7 QC read with the same identity (96%) and 90% coverage, but in third place. Panel b (bottom) shows a different alignment; here the SHI7 QC read (right) finds the same best match as the non-QC read (left), but at higher identity and lower E value (SHI7, 96% and E value of 8e−72; non-QC, 94% and E value of 1e−32). The case demonstrated by panel a occurs less frequently than panel b for this test data but may have additional important implications for pipelines relying on “best-match” read mapping.

These results are interesting in that it was not obvious that there was specific adaptor contamination (albeit at low level), nor was it obvious that a majority of these 100-bp paired-end shotgun reads overlapped throughout half their length. Downstream analyses also benefit from this procedure in straightforward ways. By way of simple illustration, matched sequences pre- and post-QC from the same HMP sample were submitted to nucleotide BLAST (9, 10) against the “nt” database. As would be expected, comparison between the stitched, quality-controlled sequences produced by SHI7 and the corresponding raw R1 reads shows higher E values after QC. In some cases, the post-QC reads received higher match identity for the same query (Fig. 3a), and in other cases a different, and presumably more probable, highest-scoring match (Fig. 3b), although this particular query may still not resolve at the subspecies level with appropriately high similarity, possibly due to the lack of its specific matching reference strain in the database. This illustrates the potential implications of QC for pipelines and analyses relying on such “best-match” alignments. Higher scores (and/or lower E values) are generally expected following stitching because longer reads have higher information content (are less likely to match longer series of nucleotides by chance), and consensus quality scores in overlapping regions are likely to result in fewer technical errors, raising match identity. The benefit in using QC to trim adaptor contamination is expected to be higher in end-to-end alignments than in local alignments, which implicitly perform soft clipping.

SHI7 was also evaluated on 16S amplicon data. A sample from the Primate Microbiome Project (11) was run through “learning mode,” which determined it contained Nextera adaptor contamination, stitchable reads (over 97% stitched successfully) with combined lengths concentrated around 252 bp. SHI7 detected that the sample was an amplicon sample due to the low coefficient of variation in the stitching sizes (<0.03). This in turn informed SHI7 to impose a length minimum and maximum near the peak to reduce contaminant reads (Fig. 2b), which we found in this sample to be primarily PhiX174 control sequences that this particular Illumina sequencing technology is known to introduce. The final run took 6 s for this sample, consisting of a 56-MB pair of input FASTQ files.

The learning module was also tested on a variety of data sets to ascertain whether it was able to discover reasonable QC parameters across sequencing protocols, technologies, and data sources. As summarized in Table 1, in each case SHI7 learns parameters that agree with expectations given background knowledge of the data sets. Some exceptions to our expectations were produced by the learning module, including the ability to stitch reads unexpectedly in the HMP tongue shotgun data, no detection of expected Nextera adaptors in the mouse tutorial data, and detection of TruSeq3-2 adaptors when ScriptSeq adaptors were used for the transcriptome sequencing (RNA-Seq) data. However, these decisions were justifiable when each case was investigated carefully: the majority of HMP tongue shotgun reads do indeed stitch appropriately, the mouse tutorial data had its forward adaptors already removed and used longer amplicons without bleed-through of reverse adapters, and the appropriate ScriptSeq adaptors were actually contained within Trimmomatic’s version of the TruSeq3-2 adaptor library.

**TABLE 1:** SHI7 learning module produces meaningful QC parametrizations on internal and publicly available data sets

Data set	Availability	Learned parameters
HMP tongue; shotgun (Illumina HS PE TS2)	Public, SRS014271 (8)	--adaptor TruSeq2 --flash True --allow_outies False --filter_qual 36 --trim_qual 36
Immigrant Microbiome Project; amplicon (mixed Illumina PE Nextera)	Internal	--adaptor Nextera --flash True --allow_outies False --filter_qual 34 --trim_qual 32
Small bowel aspirate; amplicon (Illumina PE Nextera)	Internal	--adaptor Nextera --flash True --allow_outies False --filter_qual 36 --trim_qual 34
Primate Microbiome Project stomach; amplicon (Illumina PE TS2)	Internal	--adaptor TruSeq2 --flash True --allow_outies False --filter_qual 36 --trim_qual 33 --min_overlap 239 --max_overlap 269
Longitudinal diet study; shotgun (Illumina HS SE Nextera)	Internal	-SE --adaptor Nextera --flash False --allow_outies False --filter_qual 36 --trim_qual 34
HMP stool; amplicon (454 SE)^a^	Public, stool (17)	-SE --adaptor None --flash False --allow_outies False --filter_qual 34 --trim_qual 31
Mouse tutorial; amplicon (Illumina PE Nextera)	Public (18)	--adaptor None --flash True --allow_outies False --filter_qual 34 --trim_qual 34 --min_overlap 154 --max_overlap 172
Irritable bowel syndrome cohort; shotgun (Illumina HS SE Nextera)	Internal	-SE --adaptor Nextera --flash False --allow_outies False --filter_qual 37 --trim_qual 35
Human microbiome; RNA-Seq (Illumina HS PE ScriptSeq)	Internal	--adaptor TruSeq3-2 --flash True --allow_outies False --filter_qual 39 --trim_qual 36

^a^ *sff_extract -Q* was used for the initial conversion of .sff to .Fastq format (19).

## DISCUSSION

As mentioned previously, these results are not intended to illustrate any advantage in quality or QC performance SHI7 might be expected to achieve over alternative quality control pipelines. This is particularly the case compared to pipelines developed and tested in the context of well-defined workflows utilizing known extraction, amplification, size selection, and sequencing protocols, where each step is carefully translated into the appropriate QC parameters and tested with a variety of tools against mock data sets produced by the same. SHI7 is not intended to replace such highly specialized workflows. Furthermore, regardless of the validity of the selected parameters, we strongly caution against any blind application of bioinformatics analysis tools without a working comprehension of the concepts underlying biological sequence processing, as this may lead to erroneous analyses.

Instead, the simplicity and convenience afforded by SHI7 are our primary focus, as our results imply that SHI7 is capable of achieving reasonable quality control without the need for the user to know or supply any procedural parameters in advance. Furthermore, the resulting merged FASTA file (if this output mode is used) is immediately compatible with operational taxonomic unit (OTU) picking solutions such as NINJA-OPS and others (3, 12, 13), whose outputs are in turn compatible with statistical analyses in standard metagenomics pipelines, including QIIME (14). The intention, then, is for SHI7 to bring users, in most cases, from raw FASTQ data to sequence analysis capability in a single step without needing to know any details of the sequencing procedure or technology, while still providing reasonable quality control.

We believe the ease of use, speed, flexibility, and intelligent learning capabilities of SHI7 will be of benefit to novices and experts alike, particularly when dealing with FASTQ data from various sources where the details underlying the sequencing protocol are not well known in advance. For use with both shotgun and 16S sequencing projects, as well as on data from collaborators or online repositories, which are often accompanied by sparse methodological detail, we find that SHI7 reduces time spent adjusting and exploring settings for QC parameters, while providing consistent quality control that mirrors standard practices.

SHI7 is available as free and open source software under the AGPLv3 license. Dependencies (Trimmomatic and FLASH) are distributed with the compatible GPLv3 license. The software is freely available for multiple operating systems on GitHub (15) at https://github.com/knights-lab/shi7 (see the release page for the portable package). This GitHub page also includes tutorials, example use cases, and an interface for requesting new features and filing bug reports. SHI7 may be also installed using Anaconda (https://anaconda.org/knights-lab/shi7).

## MATERIALS AND METHODS

We have developed a pipeline that integrates standard QC practices with a learning module that automatically tests for and optimizes parameters based on the sequence data itself. Each phase of the pipeline is aware of the parameters selected in other phases and optimized accordingly. This pipeline is applicable across domains, handles a range of short-read sequencing lengths, and can automatically determine the following: whether reads are likely to be paired end, whether pairs can be stitched (and with how much overlap), whether the sequences derive from amplicon or whole-genome shotgun sources, what adaptors were used on the reads (Illumina platform), with what aggressiveness to perform quality trimming, and how to transform sample/lane names into standards-compliant FASTA labels if a combined FASTA is desired (or the file names of the final FASTQ files, if FASTQ is desired as the output).

To accomplish these objectives, SHI7 incorporates just two well-known, lightweight programs, Trimmomatic (5) and FLASH (6), and introduces its own high-performance error-correcting barcode demultiplexer (gotta_split) and quality control (shi7_trimmer) modules. These new modules are written in C, and their performance saturates with the write speeds of modern hard drives. The gotta_split demultiplexer features ambiguous base support in the barcodes (including barcodes beginning with a series of “N” bases), supports staggered barcodes, barcodes occurring elsewhere in the reads than the beginning (disabled by default), and error correction up to a user-specified number of mismatches, including the ability to report whether the specified number of mismatches could cause one adaptor to be mistaken for another. The shi7_trimmer module implements numerous simple trimming methods, uses different quality cutoffs for trimming either end of the read, and filters for length and average PHRED score.

The primary reason for including the shi7_trimmer module is its variable-length sliding quality floor mode for trimming the ends of reads. This functionality is currently not available in Trimmomatic. Unlike averaged quality scores, which are commonly used in sliding-window-based quality control, the floor function will not tolerate the presence of even a single base of lower quality than a given threshold, regardless of the quality of other bases in the window. Only once all bases in in the sliding window are above this threshold will it stop trimming from the ends of a given read. This behavior is especially useful for removing residual adaptor contamination following full-read-length stitching (as for bacterial 16S V4 amplicon sequencing), where the amplified region is shorter than the technical read length, causing the resulting sequences to contain part of the opposing adaptor. Due to the merging process, these artifactual portions will likely produce poor base matching (as the forward and reverse adaptors are essentially being overlaid), which the FLASH software reports in the form of very-low-quality scores, allowing shi7_trimmer to remove them from the read.

### Interactive mode (manual selection of parameters).

SHI7 can be run without a learning mode, in which case it becomes a simplified wrapper script for standard QC practices with sensible defaults for most paired-end adaptor-free workloads assuming the possibility of stitching. Each stage of the pipeline can be turned off or on with a single command line flag (including disabling stitching, paired-end mode, combining results into FASTA, adaptor trimming with specified adaptors, or splitting FASTQ files into samples based on barcodes). Although easy to use, this mode of operation is more suitable for users with knowledge of their data generation processes, as it depends on the correct assumptions about the data: e.g., are reads paired, and if so, are they amenable to stitching, and were adaptors removed and if not, which ones? It also exposes a few command line options for each of these stages, allowing for flexible, but not overwhelming, parameter customization. Individual steps in the pipeline can also be run separately without the python wrapper, including the two C modules (gotta_split and shi7_trimmer) for even more flexibility.

### Learning mode (automatic).

The SHI7 pipeline in “learning” operation mode first applies heuristics to determine basic features of the data. These include PHRED scale determination, whether reads are paired end (using file name string pattern checks), and approximate read lengths prior to adaptor removal or quality trimming. The presence of an oligonucleotide file (text file implementing MOTHUR format paired barcodes, typically “oligos.txt”) signals to split a single pair of FASTQ files into multiple separate FASTQ files named by corresponding sample ID (16). The software also determines whether reads stitch, and if so, at what level of overlap if reads are detected to be amplicons. The quality scores at which to filter and trim reads are also learned by profiling the distribution of base qualities in a sampling of reads.

### Learning mode operation.

In its “learning mode,” SHI7 runs a learning pass on a subsampled selection of the reads across files (up to 1,000 reads per FASTQ file), gathering data by running various combinations of settings, and reports its best estimation for these parameters to the user before proceeding with the full QC pipeline using these options. Specifically, the learning module first subsamples each FASTQ file to 1,000 sequences, recording sequence lengths. To determine whether pairs are present, if an even number of FASTQ files exist, these are run through basic pattern recognition to identify if a known pattern exists across all files that successfully distinguishes pairs (“R1/R2,” “0.1/0.2,” and “_1/_2” are checked for among others in a growing list of patterns). If pairs are detected, the subsequent adaptor detection stage proceeds in paired mode (otherwise, the unpaired mode is used). The adaptor detection runs a separate instance of Trimmomatic using each one of its included repertoire of adapters and picks the adaptor set that produces the smallest output file size.

Following adaptor detection and removal, if paired-end reads are present stitching is attempted using generous defaults (minimum overlap of 10 bases and maximum overlap of 700 bases). A histogram of resulting overlaps is generated with FLASH, allowing SHI7 to determine whether a reasonable proportion of reads reliably stitch (25% or more, by default) and further assessing whether the coefficient of variation (CV) in stitched read lengths is less than 0.1, signaling significant DNA fragment length uniformity indicative of amplicon or amplicon-like reads. This allows SHI7 to “bound” the minimum and maximum overlap considered in the stitching process over an expected range (set by default to ±twice the standard deviation [SD]). This “bounded stitching” itself serves as an additional quality control agent by eliminating falsely stitched reads that result from unexpectedly long or short contaminants and reducing rare instances of tied equal scoring overlaps by restricting to overlaps in the expected range.

The final trimming quality parameters are determined by scanning the reads again (after all previous QC steps have been completed) to determine average quality as well as “terminus” quality (quality scores averaged over the first and last 10 bases of each read). The learning module recommends a per-read average quality filter equal to the average base quality throughout the data set and produces a recommendation for end trimming between this value and the average “terminus” quality calculated previously.

### Limitations.

Notable limitations of this software include reliance on Trimmomatic’s adaptor collection for detecting explicit adaptor contamination, although any adaptors can be added to this collection by the user if this information is known through the corresponding Trimmomatic interfaces. The pipeline requires both Python 2.7+ (including 3.x, for the wrapper and learning module) and Java, SE (for Trimmomatic’s adaptor removal). Minimum run time requirements include a 64-bit operating system (Windows, Linux, or OSX), 4 GB RAM (with 1 thread; add 4 GB per additional thread used), and free disk space equal to about twice the original size of the data being processed. FASTQ files must not contain entries split across lines (word wrap), and paired ends (if used) must be in split-file format. FASTQ files appearing in interleaved format (both pairs appear in the same file) are not explicitly supported in paired-end mode but will still be processed normally as though they were single-end reads. Compressed FASTQ files are not supported: the user must currently extract these files to use them with SHI7, such as with the command “gunzip *,” but support for compressed formats is planned for a future release. If demultiplexing is desired, a text file named “oligos.txt” is required in the input directory in MOTHUR format; there is no automatic detection of barcodes for demultiplexing.

### Data availability.

All code used in SHI7 is available in its repository located at https://github.com/knights-lab/shi7. External test datasets are available from their respective citations in Table 1.

### Accession number(s).

Our own validation datasets are made publicly available in the Sequence Read Archive under accession no. SRP132961.

## References

[1] Kahvejian A, Quackenbush J, Thompson JF 2008 What would you do if you could sequence everything? Nat Biotechnol 26:1125–1133. doi:10.1038/nbt1494. 18846086 PMC4153598

[2] Kuczynski J, Lauber CL, Walters WA, Parfrey LW, Clemente JC, Gevers D, Knight R 2011 Experimental and analytical tools for studying the human microbiome. Nat Rev Genet 13:47–58. doi:10.1038/nrg3129. 22179717 PMC5119550

[3] Langmead B, Salzberg SL 2012 Fast gapped-read alignment with Bowtie 2. Nat Methods 9:357–359. doi:10.1038/nmeth.1923. 22388286 PMC3322381

[4] Hamady M, Walker JJ, Harris JK, Gold NJ, Knight R 2008 Error-correcting barcoded primers allow hundreds of samples to be pyrosequenced in multiplex. Nat Methods 5:235–237. doi:10.1038/nmeth.1184. 18264105 PMC3439997

[5] Bolger AM, Lohse M, Usadel B 2014 Trimmomatic: a flexible trimmer for Illumina sequence data. Bioinformatics 30:2114–2120. doi:10.1093/bioinformatics/btu170. 24695404 PMC4103590

[6] Magoč T, Salzberg SL 2011 FLASH: fast length adjustment of short reads to improve genome assemblies. Bioinformatics 27:2957–2963. doi:10.1093/bioinformatics/btr507. 21903629 PMC3198573

[7] Kuczynski J, Stombaugh J, Walters WA, González A, Caporaso JG, Knight R 2012 Using QIIME to analyze 16S rRNA gene sequences from microbial communities. Curr Protoc Microbiol Chapter 1:Unit 1E.5. doi:10.1002/9780471729259.mc01e05s27. PMC4477843 23184592

[8] Human Microbiome Project 2012 Structure, function and diversity of the healthy human microbiome. Nature 486:207–214. doi:10.1038/nature11234. 22699609 PMC3564958

[9] Altschul SF, Gish W, Miller W, Myers EW, Lipman DJ 1990 Basic local alignment search tool. J Mol Biol 215:403–410. doi:10.1016/S0022-2836(05)80360-2. 2231712

[10] Johnson M, Zaretskaya I, Raytselis Y, Merezhuk Y, McGinnis S, Madden TL 2008 NCBI BLAST: a better web interface. Nucleic Acids Res 36(Web server issue):W5–W9. doi:10.1093/nar/gkn201. 18440982 PMC2447716

[11] Clayton JB, Vangay P, Huang H, Ward T, Hillmann BM, Al-Ghalith GA, Travis DA, Long HT, Tuan BV, Minh VV, Cabana F, Nadler T, Toddes B, Murphy T, Glander KE, Johnson TJ, Knights D 2016 Captivity humanizes the primate microbiome. Proc Natl Acad Sci U S A 113:10376–10381. doi:10.1073/pnas.1521835113. 27573830 PMC5027417

[12] Al-Ghalith GA, Montassier E, Ward HN, Knights D 2016 NINJA-OPS: fast accurate marker gene alignment using concatenated ribosomes. PLoS Comput Biol 12:e1004658. doi:10.1371/journal.pcbi.1004658. 26820746 PMC4731464

[13] Edgar RC 2010 Search and clustering orders of magnitude faster than BLAST. Bioinformatics 26:2460–2461. doi:10.1093/bioinformatics/btq461. 20709691

[14] Caporaso JG, Kuczynski J, Stombaugh J, Bittinger K, Bushman FD, Costello EK, Fierer N, Peña AG, Goodrich JK, Gordon JI, Huttley GA, Kelley ST, Knights D, Koenig JE, Ley RE, Lozupone CA, McDonald D, Muegge BD, Pirrung M, Reeder J, Sevinsky JR, Turnbaugh PJ, Walters WA, Widmann J, Yatsunenko T, Zaneveld J, Knight R 2010 QIIME allows analysis of high-throughput community sequencing data. Nat Methods 7:335–336. doi:10.1038/nmeth.f.303. 20383131 PMC3156573

[15] Al-Ghalith G, Hillmann B, Ang K, Shields-Cutler R, Knights D 2017 SHI7: a streamlined short-read iterative trimming pipeline. Zenodo doi:10.5281/zenodo.1009163.

[16] Schloss PD, et al. 30 11 2015 Oligos file—Mothur. https://www.mothur.org/wiki/Oligos_File.

[17] Turnbaugh PJ, Ley RE, Hamady M, Fraser-Liggett CM, Knight R, Gordon JI 2007 The Human Microbiome Project: exploring the microbial part of ourselves in a changing world. Nature 449:804–810. doi:10.1038/nature06244. 17943116 PMC3709439

[18] Comeau AM, Douglas GM, Langille MG 2017 Microbiome helper: a custom and streamlined workflow for microbiome research. mSystems 2:e00127-16. doi:10.1128/mSystems.00127-16. PMC5209531 28066818

[19] Blanca J 2017 seq_crumbs: little sequence file utilities meant to work within UniX pipelines. GitHub https://github.com/JoseBlanca/seq_crumbs.

